# A Note on an Improved Self-Healing Group Key Distribution Scheme

**DOI:** 10.3390/s151025033

**Published:** 2015-09-29

**Authors:** Hua Guo, Yandong Zheng, Biao Wang, Zhoujun Li

**Affiliations:** 1 State Key Laboratory of Software Development Environment, Beihang University, Beijing 100191, China; E-Mail: lizj@buaa.edu.cn; 2 Beijing Key Laboratory of Network Technology, Beihang University, Beijing 100191, China; E-Mail: zy1406423@buaa.edu.cn; 3 School of Information Science and Technology, University of International Relations, Beijing 100091, China; E-Mail: wangbiao@uir.edu.cn

**Keywords:** self-healing group key distribution, forward security, backward secrecy, collusion attack

## Abstract

In 2014, Chen *et al.* proposed a one-way hash self-healing group key distribution scheme for resource-constrained wireless networks in the journal of Sensors (14(14):24358-24380, doi: 10.3390/s141224358). They asserted that their Scheme 2 achieves mt-revocation capability, mt-wise forward secrecy, any-wise backward secrecy and has mt-wise collusion attack resistance capability. Unfortunately, this paper pointed out that their scheme does not satisfy the forward security, mt-revocation capability and mt-wise collusion attack resistance capability.

## 1. Introduction

Group communication includes a group manager (GM) and some group members, in which all of the group members share a common session key which is distributed by GM. In order to achieve secure group communication in unreliable wireless networks, Staddon *et al.* [1] introduced a group key distribution scheme with self-healing mechanism, which allows a group member to recover session keys even if he doesn’t receive the corresponding broadcast messages because of packet loss, without requesting anything to the group manager. Recently, Chen *et al.* [2] developed two schemes to realize the self-healing group key distribution based on one-way hash chain. The proposed Scheme 2 has the constant storage overhead and low communication overhead, thus is very suitable for the resource-constrained wireless networks. They assert that their scheme is secure, *i.e.*, satisfies mt-revocation capability, mt-wise forward secrecy, any-wise backward secrecy and resistance to mt-wise collusion attack. Unfortunately, we found a revoked user can recover other legitimate users’ personal secrets which can be used to recover the current session’s session key, this directly breaks the forward security, mt-revocation capability and mt-wise collusion attack resistance capability. Thus, Chen *et al.*’s Scheme 2 is insecure.

## 2. Overview of Chen *et al.*’s Scheme

Chen *et al.*’s self-healing group key distribution Scheme 2 includes five parts: Set up, Broadcast in session *j*, Group session key recovery and self-healing, Group member addition and Group member revocation. Here we only describe the first three parts which is helpful to understand the attack.

(1) Set up
  The GM selects a random 2*t*-degree polynomial $s_{1}\left(x\right)=a_{0}+a_{1}x+\cdots +a_{2t}x^{2t}$ and a random *t*-degree polynomial $s_{2}\left(x\right)=b_{0}+b_{1}x+\cdots +b_{t}x^{t}$ from $F_{q}\left[x\right]$. Then, the GM chooses a random value $\varepsilon_{1}$ from $F_{q}$. The GM sends the user’s personal secret $S_{i}=\left\{\varepsilon_{1}\cdot s_{1}\left(i\right),\varepsilon_{1}\cdot s_{2}\left(i\right)\right\}$ to a user via a secure channel.
(2) Broadcast in session *j* (for 1≤j≤m)
  Let $R_{j}=\left\{R_{j}^{1},R_{j}^{2},\cdots ,R_{j}^{j^{\prime }},\cdots ,R_{j}^{j}\right\}$ be the set of revoked users before and in session *j*, where $R_{j}^{j^{\prime }}$ is the set of users who join the group in session $j^{\prime }$ and are revoked before and in session *j*. $R_{j}^{j^{\prime }}=\left\{U_{r_{1}^{j^{\prime }}},U_{r_{2}^{j^{\prime }}},\cdots ,U_{r_{w_{j^{\prime }}}^{j^{\prime }}}\right\}$ and $|R_{j}^{j^{\prime }}|=w_{j^{\prime }}\leq t$. $r_{1}^{j^{\prime }},r_{2}^{j^{\prime }},\cdots ,r_{w_{j^{\prime }}}^{j^{\prime }}$ are the IDs of users in $R_{j}^{j^{\prime }}$. $R_{j}^{j^{\prime }}=\emptyset$ if no users joined the group in session $j^{\prime }$.
  - The GM chooses a random value $k_{j}^{0}\in F_{q}$ and a one-way hash function h(·). Note that $h^{i}\left(\cdot \right)$ denotes applying *i* times hash operation. Then GM constructs the *j*-th key chain for session *j*: $\left\{k_{j}^{1},k_{j}^{2},\cdots ,k_{j}^{j}\right\}$, where
    $$\begin{array}{ccc} k_{j}^{1} & = & h(k_{j}^{0})\\ k_{j}^{2} & = & h\left(k_{j}^{1}\right)=h\left(h\left(k_{j}^{0}\right)\right)=h^{2}\left(k_{j}^{0}\right)\\ & \cdots , & \\ k_{j}^{j} & = & h\left(k_{j}^{j-1}\right)=h\left(h\left(k_{j}^{j-2}\right)\right)=\cdots =h^{j}\left(k_{j}^{0}\right) \end{array}$$
    For security, $k_{j}^{0}\left(1\leq j\leq m\right)$ is different from each other.
    The GM splits the $k_{j}^{j^{\prime }}$ into two *t*-degree polynomials, $U_{j}^{j^{\prime }}\left(x\right)$ and $V_{j}^{j^{\prime }}\left(x\right)$, where
    $$k_{j}^{j^{\prime }}=U_{j}^{j^{\prime }}\left(x\right)+V_{j}^{j^{\prime }}\left(x\right),j^{\prime }=1,2,\cdots ,j$$
  - To construct the revocation polynomials for session *j*, the GM firstly chooses number sets ${\bar{R}}_{j}^{j^{\prime }}$, where ${\bar{R}}_{j}^{j^{\prime }}=\left\{{\bar{r}}_{1}^{j^{\prime }},{\bar{r}}_{2}^{j^{\prime }},\cdots ,{\bar{r}}_{t-w_{j^{\prime }}}^{j^{\prime }}\right\}$ are random numbers which are not used as a user ID and different from each other. Then, the GM computes
    $$A_{j}^{j^{\prime }}\left(x\right)=\Pi_{z=1}^{|R_{j}^{j^{\prime }}|}\left(x-r_{z}^{j^{\prime }}\right)\Pi_{z^{\prime }=1}^{t-|R_{j}^{j^{\prime }}|}\left(x-{\bar{r}}_{z^{\prime }}^{j^{\prime }}\right),j^{\prime }=1,2,\cdots ,j$$
  - The GM chooses a random session key $K_{j}$ from $F_{q}$. Then, the GM computes
    $$M_{j}^{j^{\prime }}\left(x\right)=A_{j}^{j^{\prime }}\left(x\right)\cdot U_{j}^{j^{\prime }}\left(x\right)+\varepsilon_{j^{\prime }}\cdot s_{1}\left(x\right)$$
    and
    $$N_{j}^{j^{\prime }}\left(x\right)=V_{j}^{j^{\prime }}\left(x\right)+\varepsilon_{j^{\prime }}\cdot s_{2}\left(x\right)$$
    After that, the GM broadcasts the message
    $$\begin{array}{ccc} B_{j} & = & R_{j}\cup {\bar{R}}_{j}\cup \left\{M_{j}^{j^{\prime }}\left(x\right)|j^{\prime }=1,2,\cdots ,j\right\}\cup \left\{N_{j}^{j^{\prime }}\left(x\right)|j^{\prime }=1,2,\cdots ,j\right\}\\ & & \cup \{E_{k_{j}^{j^{\prime }}}\left(K_{j^{\prime }}\right)|j^{\prime }=1,2,\cdots ,j\} \end{array}$$
    where ${\bar{R}}_{j}=\left\{{\bar{R}}_{j}^{1},{\bar{R}}_{j}^{2},\cdots ,{\bar{R}}_{j}^{j}\right\}$ and $E_{k}\left(\cdot \right)$ is a symmetric encryption function.
(3) Group session key recovery and self-healing
  Any legitimate user $U_{i}\in G_{j}^{j^{\prime }}$ can recover the *j*-th session key when he receives the broadcast message $B_{j}$ as follows.
  - $U_{i}$ uses his personal secret $\varepsilon_{j^{\prime }}\cdot s_{1}\left(i\right)$ and $\varepsilon_{j^{\prime }}\cdot s_{2}\left(i\right)$ to compute
    $$U_{j}^{j^{\prime }}\left(i\right)=\frac{M_{j}^{j^{\prime }}\left(i\right)-\varepsilon_{j^{\prime }}\cdot s_{1}\left(i\right)}{A_{j}^{j^{\prime }}\left(i\right)}$$
    and
    $$V_{j}^{j^{\prime }}\left(i\right)=N_{j}^{j^{\prime }}\left(i\right)-\varepsilon_{j^{\prime }}\cdot s_{2}\left(i\right)$$
    Then, $U_{i}$ computes $k_{j}^{j^{\prime }}=U_{j}^{j^{\prime }}\left(i\right)+V_{j}^{j^{\prime }}\left(i\right)$.
  - $U_{i}$ uses the hash function h(·) to compute all $\left\{k_{j}^{j^{{}^{\prime \prime }}}\right\}$ for $j^{\prime }<j^{{}^{\prime \prime }}\leq j$ in the *j*-th key chain.
  - $U_{i}$ recovers the session keys $\left\{K_{j^{{}^{\prime \prime }}}\right\}\left(j^{\prime }<j^{{}^{\prime \prime }}\leq j\right)$ by decrypting $E_{k_{j}^{j^{{}^{\prime \prime }}}}\left(K_{j^{{}^{\prime \prime }}}\right)$$\left(j^{\prime }<j^{{}^{\prime \prime }}\leq j\right)$ with corresponding keys $\left\{k_{j}^{j^{{}^{\prime \prime }}}\right\}\left(j^{\prime }<j^{{}^{\prime \prime }}\leq j\right)$.

## 3. Cryptanalysis of Chen *et al.*’s Scheme 2

In this section we exhibit the attack on Chen *et al.*’s Scheme 2 step by step, and explain why this attack exists.

### 3.1. Attack on Chen et al.’s Scheme 2

Let $G_{j_{1}}^{j^{\prime }}$ denote the users who join the group in session $j^{\prime }$ and are still legitimate in session $j_{1}$ where $j^{\prime }<j_{1}$. Suppose that $U_{i}\in G_{j_{1}}^{j^{\prime }}$ and $U_{i}$ is revoked in session $j_{2}\left(j^{\prime }<j_{1}<j_{2}\right)$. Now we are ready to show how $U_{i}$, who is revoked in session $j_{2}$, recovers the personal secret of another user who is legitimate in session $j_{2}$, furthermore uses this personal secret to compute the session key $K_{j_{2}}$ which should be kept secret from $U_{i}$.

**Step 1.** $U_{i}$ computes $k_{j^{\prime }}^{j{}^{\prime }}$ and $k_{j_{1}}^{j^{\prime }}$ with his personal key $S_{i}$ and the broadcast messages $M_{j^{\prime }}^{j^{\prime }}\left(x\right)$, $N_{j^{\prime }}^{j^{\prime }}\left(x\right)$ and $M_{j_{1}}^{j^{\prime }}\left(x\right)$, $N_{j_{1}}^{j^{\prime }}\left(x\right)$.
**Step 2.** In session $j^{\prime }$, $U_{i}$ receives the broadcast messages $M_{j^{\prime }}^{j^{\prime }}\left(x\right),N_{j^{\prime }}^{j^{\prime }}\left(x\right)$, where
  (1) $$\begin{array}{c} M_{j}^{j^{\prime }}\left(x\right)=A_{j}^{j^{\prime }}\left(x\right)\cdot U_{j}^{j^{\prime }}\left(x\right)+\varepsilon_{j^{\prime }}\cdot s_{1}\left(x\right) \end{array}$$
  and
  (2) $$\begin{array}{c} N_{j}^{j^{\prime }}\left(x\right)=V_{j}^{j^{\prime }}\left(x\right)+\varepsilon_{j^{\prime }}\cdot s_{2}\left(x\right) \end{array}$$
  Note that $k_{j^{\prime }}^{j^{\prime }}=U_{j^{\prime }}^{j^{\prime }}\left(x\right)+V_{j^{\prime }}^{j^{\prime }}\left(x\right)$, Equation (2) can be converted to $N_{j}^{j^{\prime }}\left(x\right)=k_{j^{\prime }}^{j^{\prime }}-U_{j}^{j^{\prime }}\left(x\right)+\varepsilon_{j^{\prime }}\cdot s_{2}\left(x\right)$.
  Let Equation (1) + $A_{j}^{j^{\prime }}\left(x\right)\cdot Equation\left(2\right)$, $U_{i}$ can obtain
  (3) $$\begin{array}{c} M_{j^{\prime }}^{j^{\prime }}\left(x\right)+A_{j^{\prime }}^{j^{\prime }}\left(x\right)\cdot N_{j^{\prime }}^{j^{\prime }}\left(x\right)=k_{j^{\prime }}^{j^{\prime }}\cdot A_{j^{\prime }}^{j^{\prime }}\left(x\right)+\varepsilon_{j^{\prime }}\cdot s_{1}\left(x\right)+A_{j^{\prime }}^{j^{\prime }}\left(x\right)\cdot \varepsilon_{j^{\prime }}\cdot s_{2}\left(x\right) \end{array}$$
  With the values of $k_{j^{\prime }}^{j^{\prime }}$ which is computed from step (1), $U_{i}$ can obtain
  (4) $$\begin{array}{c} M_{j^{\prime }}^{j^{\prime }}\left(x\right)+A_{j^{\prime }}^{j^{\prime }}\left(x\right)\cdot N_{j^{\prime }}^{j^{\prime }}\left(x\right)-A_{j^{\prime }}^{j^{\prime }}\left(x\right)\cdot k_{j^{\prime }}^{j^{\prime }}=\varepsilon_{j^{\prime }}\cdot s_{1}\left(x\right)+A_{j^{\prime }}^{j^{\prime }}\left(x\right)\cdot \varepsilon_{j^{\prime }}\cdot s_{2}\left(x\right) \end{array}$$
**Step 3.** Since $U_{i}$ is legitimate in session $j_{1}$, $U_{i}$ can obtain the similar result in the same way:
  (5) $$\begin{array}{c} M_{j_{1}}^{j^{\prime }}\left(x\right)+A_{j_{1}}^{j^{\prime }}\left(x\right)\cdot N_{j_{1}}^{j^{\prime }}\left(x\right)-A_{j_{1}}^{j^{\prime }}\left(x\right)\cdot k_{j_{1}}^{j^{\prime }}=\varepsilon_{j^{\prime }}\cdot s_{1}\left(x\right)+A_{j_{1}}^{j^{\prime }}\left(x\right)\cdot \varepsilon_{j^{\prime }}\cdot s_{2}\left(x\right) \end{array}$$
  Let Equation (4) – Equation (5), user $U_{i}$ can obtain
  (6) $$\begin{array}{c} \begin{array}{cc} & M_{j^{\prime }}^{j^{\prime }}\left(x\right)+A_{j^{\prime }}^{j^{\prime }}\left(x\right)\cdot N_{j^{\prime }}^{j^{\prime }}\left(x\right)-A_{j^{\prime }}^{j^{\prime }}\left(x\right)\cdot k_{j^{\prime }}^{j^{\prime }}-M_{j_{1}}^{j^{\prime }}\left(x\right)-A_{j_{1}}^{j^{\prime }}\left(x\right)\cdot N_{j_{1}}^{j^{\prime }}\left(x\right)+A_{j_{1}}^{j^{\prime }}\left(x\right)\cdot k_{j_{1}}^{j^{\prime }}\\ = & \left(A_{j^{\prime }}^{j^{\prime }}\left(x\right)-A_{j_{1}}^{j^{\prime }}\left(x\right)\right)\cdot \varepsilon_{j^{\prime }}\cdot s_{2}\left(x\right) \end{array} \end{array}$$
**Step 4.** $U_{i}$ computes $\varepsilon_{j^{\prime }}\cdot s_{2}\left(x\right)$ as
  (7) $$\begin{array}{c} \begin{array}{cc} & \varepsilon_{j^{\prime }}\cdot s_{2}\left(x\right)\\ = & \frac{M_{j^{\prime }}^{j^{\prime }}\left(x\right)+A_{j^{\prime }}^{j^{\prime }}\left(x\right)\cdot N_{j^{\prime }}^{j^{\prime }}\left(x\right)-A_{j^{\prime }}^{j^{\prime }}\left(x\right)\cdot k_{j^{\prime }}^{j^{\prime }}-M_{j_{1}}^{j^{\prime }}\left(x\right)-A_{j_{1}}^{j^{\prime }}\left(x\right)\cdot N_{j_{1}}^{j^{\prime }}\left(x\right)+A_{j_{1}}^{j^{\prime }}\left(x\right)\cdot k_{j_{1}}^{j^{\prime }}}{\left(A_{j^{\prime }}^{j^{\prime }}\left(x\right)-A_{j_{1}}^{j^{\prime }}\left(x\right)\right)} \end{array} \end{array}$$
  Take $\varepsilon_{j^{\prime }}\cdot s_{2}\left(x\right)$ to Equation (3), $U_{i}$ computes $\varepsilon_{j^{\prime }}\cdot s_{1}\left(x\right)$ as
  (8) $$\begin{array}{c} \varepsilon_{j^{\prime }}\cdot s_{1}\left(x\right)=M_{j^{\prime }}^{j^{\prime }}\left(x\right)+A_{j^{\prime }}^{j^{\prime }}\left(x\right)\cdot N_{j^{\prime }}^{j^{\prime }}\left(x\right)-A_{j^{\prime }}^{j^{\prime }}\left(x\right)\cdot k_{j^{\prime }}^{j^{\prime }}-A_{j^{\prime }}^{j^{\prime }}\left(x\right)\cdot \varepsilon_{j^{\prime }}\cdot s_{2}\left(x\right) \end{array}$$
**Step 5.** $U_{i}$ gets a legitimate user’s identity, *v*, in session $j_{2}$ by observing $R_{j}^{j^{\prime }}$ where $j>j_{2}$.
**Step 6.** $U_{i}$ computes $\varepsilon_{j^{\prime }}\cdot s_{1}\left(v\right)$ and $\varepsilon_{j^{\prime }}\cdot s_{2}\left(v\right)$ through $\varepsilon_{j^{\prime }}\cdot s_{1}\left(x\right)$ and $\varepsilon_{j^{\prime }}\cdot s_{2}\left(x\right)$. Then, $U_{i}$ pretends $U_{v}$ to compute the session key $K_{j_{2}}$ using $\varepsilon_{j^{\prime }}\cdot s_{1}\left(v\right),\varepsilon_{j^{\prime }}\cdot s_{2}\left(v\right)$ and $M_{j_{2}}^{j^{\prime }}\left(x\right),N_{j_{2}}^{j^{\prime }}\left(x\right)$ from the broadcast message $B_{j_{2}}$.

Note that $U_{i}$ is revoked in session $j_{2}$, thus he should not have computed $K_{j_{2}}$. Therefore the scheme cannot achieve the forward security. When the revoked user $U_{i}$ obtains the session key $K_{j_{2}}$, he can of course give this session key to a new user who joins the group after session $j_{2}$ and should not know $K_{j_{2}}$. Hence, the scheme can not resist the collusion attack. Similarly, the scheme does not have the mt-revocation capability.

### 3.2. Analysis of the Weakness

Chen *et al.* [2] proposed two one-way hash chain self-healing group key distribution schemes based on the revocation polynomial in their paper. In fact, in the first scheme, each $k_{j}^{j^{\prime }}$ is masked by different masking polynomials, $\left\{\varepsilon_{j^{\prime }}\cdot s_{j}\left(x\right)|j=j^{\prime },j^{\prime }+1,\cdots ,m\right\}$, which makes the scheme to be more secure. However, Chen *et al.* claimed that using multiple masking polynomials does not contribute to the security. Based on this consideration, they presented the second scheme only using one masking polynomial for each $k_{j}^{j^{\prime }}$ to reduce the number of masking polynomials and the personal secret stored by each user. Thus the second scheme achieves the optimal storage overhead.

Now let us check the attack again. From the above attack, it is easy to find that only using one masking polynomial to construct the personal secret directly makes the Equation (6) (in step 4) hold, where $\varepsilon_{j^{\prime }}\cdot s_{1}\left(x\right)$ disappears when Equation (4) minus Equation (5). Furthermore, $\varepsilon_{j^{\prime }}\cdot s_{2}\left(x\right)$ can be computed by the revoked user $U_{i}$ through the Equation (7), which leads to the exposure of those users’ personal secret who join the group in session $j^{\prime }$, and finally results in the exposure of the session keys which should be kept secret from $U_{i}$.

Chen *et al.* [2] list Theorem 5 to show the security of their Scheme 2, thus Theorem 5 does not hold. To sum up, multiple masking polynomials should be adopted to design a secure self-healing group key distribution schemes using the polynomial secret sharing as the basic cryptographic technique. Unfortunately, multiple masking polynomials brings in the linear storage overhead. How to design a secure self-healing group key distribution schemes with constant storage overhead based on the polynomial secret sharing technique is still an open problem.

## 4. Conclusions

Chen *et al.* claimed that their self-healing group key distribution Scheme 2 achieves all basic security properties. Unfortunately, we found that Chen *et al.*’s Scheme 2 is insecure. Some security flaws are pointed out in this paper, *i.e.*, the Scheme 2 can not hold the forward security, mt-revocation capability and mt-wise collusion attack resistance capability.
